# Commentary: Slowing of the Time Course of Acidification Decreases the Acid-Sensing Ion Channel 1a Current Amplitude and Modulates Action Potential Firing in Neurons

**DOI:** 10.3389/fncel.2021.714204

**Published:** 2021-07-16

**Authors:** Matthew William, Victoria Cegielski, Xiang-Ping Chu

**Affiliations:** Department of Biomedical Sciences, School of Medicine, University of Missouri-Kansas City, Kansas City, MO, United States

**Keywords:** acid-sensing ion channel 1, slow pH change, action potential, neurodegenerative disease, patch-clamp recording

## Introduction

Multiple disorders are associated with a decrease in cerebral extracellular pH, including brain ischemia, seizures, and neurodegenerative diseases like multiple sclerosis (Vergo et al., 2011; Chu and Xiong, 2012; Friese et al., 2014; Leng et al., 2014; Tóth et al., 2020). The increase in concentration of protons that this acidic state leads to is capable of activating acid-sensing ion channels (ASICs), which are widely expressed in the nervous system (Price et al., 2014; Storozhuka et al., 2021). The most predominant of these channels in the brain is the ASIC1a subtype (Waldmann et al., 1997; Chu et al., 2014; Gründer and Pusch, 2015). Activation of ASIC1a is capable of inducing membrane depolarization in the neuron, increasing the probability of an action potential (AP) spike (Baron et al., 2002; Jiang et al., 2009; Wemmie et al., 2013; Boscardin et al., 2016). Empirically, it was well-documented how ASIC1a responds to rapid drops in pH by inducing membrane depolarization (Waldmann et al., 1997; Krishtal, 2015). Despite this, multiple conditions instead involve a slow (>10 s) decrease in pH, such as ischemia, inflammation, and seizures (Chesler, 2003). Furthermore, due to the pH dependence of the desensitization of ASICs including ASIC1a, the amplitudes of the peak current depend on the speed of acidification. For example, if the rise in proton concentration is significantly slower than desensitization, peak currents will be reduced (Gründer and Pusch, 2015). In addition, studies on the effect of ASIC activation on neuron excitability have also shown that transient depolarization induced by small pH drops (e.g., pH6.6) can trigger an initial AP as well as spontaneous AP trains in neurons, whereas lower pH values reduced AP firing and can only produce an initial AP at pH5.0. This is in good agreement with inactivation of voltage-gated sodium channels (Baron et al., 2002; Vukicevic and Kellenberger, 2004). While it is known that ASIC1a plays a critical role in reacting to such events, the extent of its response to these conditions has been relatively unknown. Recently, a team from Kellenberger's laboratory uncovered ASIC1a's response to slow changes in pH (Alijevic et al., 2020). They reported that ASIC1a leads to differing levels of neuronal activity with different rates of pH change for the same pH of activation. They provide a well-documented quantitative description of these effects, including how amplitude and speed of the pH change affect AP firing in cortical neurons, as well as the development of a Hodgkin-Huxley (HH)-based model of ASIC1a function. This demonstrates that the responses of ASIC1a to acidosis are dependent on the amount of time it takes for that change to be reached and that slow pH changes (between 4 and 10 s) also affect neuronal activation.

## Intermediately Slow pH Change Leads to Increased AP Duration

A Swiss study from *Frontiers in Cellular Neuroscience* (Alijevic et al., 2020) recently added to literature regarding the most prevalent ASIC subtype in the brain, ASIC1a. Previous studies that focused on ASIC1a reported fast pH changes (fall time <4 s) which induced membrane depolarization of this channel (Jiang et al., 2009; Kellenberger and Schild, 2015). Alijevic and other authors from Kellenberger's laboratory filled a gap in ASIC1a knowledge by researching how intermediately slow acidification (fall time between 4 and 10 s periods of pH changes) and slow acidification (fall time >10 s periods of pH changes) impacted the amplitudes of ASIC1a current and duration of the current. To do so, they recorded the ASIC1a currents in Chinese hamster ovary (CHO) cells expressing the ASIC1a protein and developed a kinetic model of this current. This kinetic model was then integrated into a HH-based neuronal model. The HH approach was chosen over a Markovian model in order to accommodate a greater number of parameters. These parameters included two types of kinetics observed in ASIC1a: rapid and slow transitions. In the experiment, outside-out patch recordings were used to test rapid transitions including open channel desensitization and deactivation, whereas slower processes like steady-state desensitization and recovery from desensitization were measured using whole-cell patch configuration. Using the HH model, they found that intermediately slow acidification of ASIC1a, falling in the 4–10 s time range called intermediately slow pH ramps, decreased ASIC1a current amplitudes, but increased neuronal action potentials (APs) with this protocol. This intermediately slow pH ramp (e.g., 5 s) produced decreased ASIC1a current amplitudes by causing APs to fire later and with higher number. The enhanced numbers of APs and firing time are likely due to increased acceleration of the kinetics of the inactivation gate of voltage-gated sodium channels (Vilin et al., 2012). In addition to this, the m-gate of voltage-gated sodium channel requires a negative shift in its voltage dependence, and the h-gate of voltage-gated sodium channel requires a positive shift in its voltage dependence to experience enhanced numbers of APs and firing time. They further examined the neuronal activation in neurons from wild-type, ASIC1a and ASIC2 knockout mice. They found that pH drop did not produce any APs regardless of acidification time in cortical neurons from ASIC1a knockout mice. However, APs recorded in neurons from wild-type and ASIC2 knockout mice were affected by slower pH ramps. The results suggest that the presence and functionality of ASIC1a plays a critical role in producing APs in cortical neurons. Furthermore, slow acidification that timed >10 s did not generate any ASIC currents regardless of neuron type in most of the experiments.

## Discussion

This new study from Kellenberger's laboratory significantly adds to the ASIC field by detailing how ASIC1a reacts to pH changes that last longer than 4 s (Alijevic et al., 2020). Because they analyze long-term pH changes in ASICs, as opposed to most other ASIC studies which utilize rapid pH change in milliseconds range (Xiong et al., 2004; Jiang et al., 2009, 2017), future studies should be conducted to confirm these findings and expand on their significance. For instance, they found that intermediate acidification of ASIC1a increases neuronal activation and decreases the ASIC1a current amplitude, but the clinical manifestations of this finding are still unknown. Future studies should be conducted to identify the effects of this increased AP duration, and how it contributes to the pathology of diseases that involve intermediately slow acidification. Additionally, the study has shown that slow acidification over 10 s leads to virtually no AP generation. Although diseases states such as ischemia involve slow acidification, ASIC1a still plays a critical role in such conditions (Xiong et al., 2004). In ischemia-induced acidosis, ASIC1a is permeable to calcium ions, leading to death of the neuron (Xiong et al., 2004; Chu and Xiong, 2012); activation of ASIC1a also can trigger necroptosis by Ca^2+^-independent mechanisms (Wang et al., 2015). While many studies have focused on the role of ASICs in neurons, not many have investigated the role that ASICs play in other cell types in the brain. For instance, ASIC1 has shown to be expressed in high-grade gliomas and was involved in tumor growth (Berdiev et al., 2003; Sheng et al., 2021). Future studies may examine how ASIC1 activated by slow pH changes in high-grade gliomas can affect the tumor growth. Thus, targeting ASIC1a becomes a promising therapeutic strategy for neurodegenerative diseases and brain tumors (Chu and Xiong, 2012; Chu et al., 2014).

## Author Contributions

All authors listed have made a substantial, direct and intellectual contribution to the work, and approved it for publication.

## Conflict of Interest

The authors declare that the research was conducted in the absence of any commercial or financial relationships that could be construed as a potential conflict of interest.
